# The challenge of therapeutic adherence in patients with first-episode psychosis: The rise of pharmacogenetics

**DOI:** 10.1192/j.eurpsy.2025.1822

**Published:** 2025-08-26

**Authors:** C. García Cerdán, P. Salas Aranda, C. Martín Gómez, C. Lorenzo Romo, M. C. Turrión Gómez, M. Isidoro García, J. Pérez

**Affiliations:** 1 Psychiatry, Salamanca University Healthcare Complex; 2 Unit of Psychiatry and Neuroscience, Institute Of Biomedical Research of Salamanca (IBSAL); 3 Medicine, University of Salamanca; 4 Biochemistry and Clinical Analysis, Salamanca University Healthcare Complex; 5 Pharmacogenetics and Precision Medicine Unit, Institute Of Biomedical Research of Salamanca (IBSAL), Salamanca, Spain

## Abstract

**Introduction:**

During the last decades, clinical practice for patients with first-episode psychosis has shifted to early intervention programmes that aim to accelerate access to treatment and also ensure adherence to antipsychotic (AP) medication. However, recent pragmatic, randomised controlled trials still report very high discontinuation rates of the initially prescribed AP treatment due to lack of efficacy, side effects, or other reasons. The CLinical Utility of early intervention including the 5-Step Precision Medicine (5SPM) Method in first-episode Psychosis (CLUMP) Project is a translational research initiative focused on improving adherence to antipsychotic medications and therapeutic outcomes in first-episode psychosis patients using pharmacogenetics. We present a preliminary descriptive analysis of this project based on a retrospective cohort.

**Objectives:**

- To establish discontinuation rates of the first antipsychotic treatment administered to patients with a first-episode psychosis before the implementation of the CLUMP project.
- To describe reasons for such discontinuations.

**Methods:**

We conducted an observational study on a consecutive, retrospective cohort of 49 patients who experienced a first-episode psychosis in Salamanca, Spain, before the implementation of CLUMP, in order to subsequently determine the impact this project might have on AP treatment discontinuations. We reviewed their clinical records to collect variables such as sex, age, first prescribed antipsychotic medication, and any discontinuation within the first year post-treatment initiation, recording reasons for discontinuation or treatment changes, such as lack of efficacy, non-adherence, side effects, or others.

**Results:**

Of the 49 patients included in the analysis, 7 were excluded due to inaccessible information, leaving a final sample of 42 (26 males). Age ranged between 16 and 48 years, with a mean age of 25.9 (9.2). 38 patients (90.47%) discontinued treatment within one year, with similar rates in men (92.3%) and women (87.5%) (See Figure 1). 16 reported side effects as the main reason for discontinuation, 1 lack of efficacy, 9 lack of adherence, and 12 other reasons (Figure 2). Among the 16 who discontinued due to side effects (Figure 3), 3 experienced extrapyramidal symptoms, 3 drowsiness, 3 sexual dysfunction, 2 hyperprolactinemia/galactorrhea, 1 metabolic syndrome, 1 excessive salivation, and 3 did not specify the symptom.

**Image:**

**Figure:**
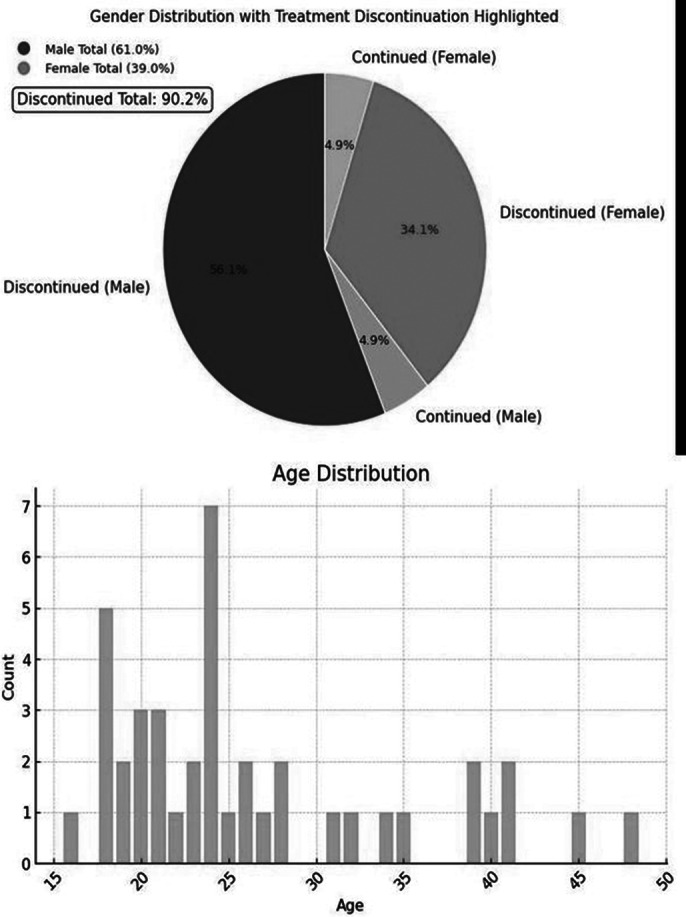

**Image 2:**

**Figure:**
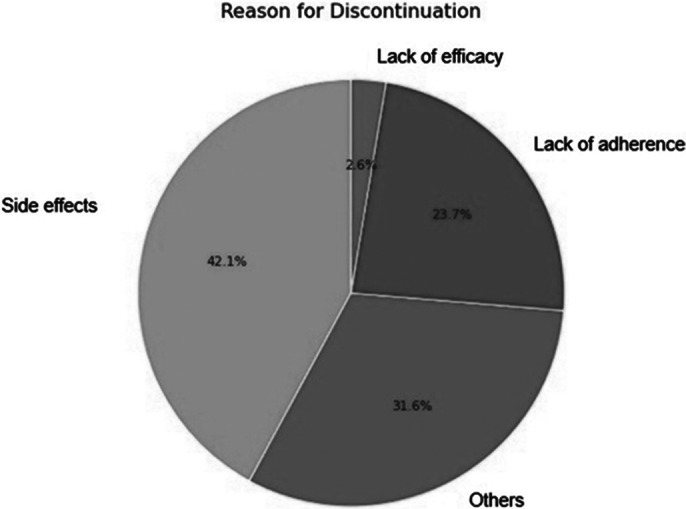

**Image 3:**

**Figure:**
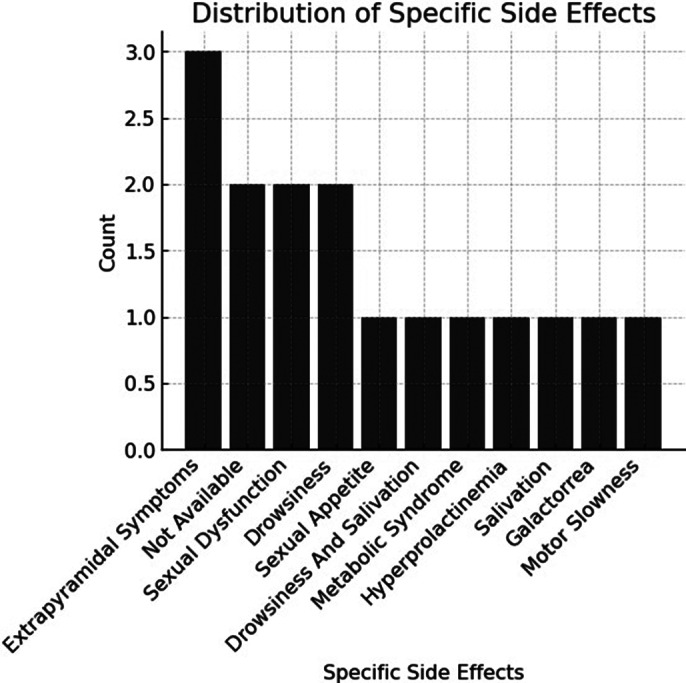

**Conclusions:**

Most patients in our sample discontinued antipsychotic treatment within the first year post-treatment initiation, mainly due to side effects. These data indicate the need to restructure clinical care for patients with first-episode psychosis to ensure adherence to AP and reduce trial-error approaches to treatment choice from start. The CLUMP project proposes an individualised strategy, based on pharmacogenetics, to improve therapeutic adherence and outcomes in patients with FEP.

**Disclosure of Interest:**

None Declared

